# Supplementary material for: Stable isotope analyses identify trophic niche partitioning between sympatric terrestrial vertebrates in coastal saltmarshes with differing oiling histories
Source: PeerJ. 2021 Jul 16;9:e11392. doi: 10.7717/peerj.11392 (PMC8288111; doi:10.7717/peerj.11392)
Supplement: Supplemental Information 1 — Modal (95% CI) trophic positions (TP) and posterior alpha [estimate of importance of terrestrial food] of sympatric seaside sparrow (A. maritimus) and marsh rice rat (O. palustris) at oiled and unoiled sites (n = samples sizes at each site) assuming terrestrial and aquatic baselines. Different letters indicate significant differences in each column (PP > 0.95) based on the probability associated with paired comparisons for species at each site in each year. [file peerj-09-11392-s001.docx]

**Table S1:** Modal (95% CI) trophic positions (TP) and posterior alpha [estimate of importance of terrestrial food] of sympatric seaside sparrow (A. maritimus) and marsh rice rat (O. palustris) at oiled and unoiled sites (n = samples sizes at each site) assuming terrestrial and aquatic baselines. Different letters indicate significant differences in each column (PP > 0.95) based on the probability associated with paired comparisons for species at each site in each year.

| Year |  | **2015** | | | **2016** | | | **2017** | | |
| --- | --- | --- | --- | --- | --- | --- | --- | --- | --- | --- |
| Species | Site | n | TP | α | n | TP | α | n | TP | α |
| *Rats* | **O1** | 7 | 3.1 (2.9-3.4)^a^ | 0.7 (0.7-0.8)^a^ | 4 | 2.7 (2.3-3.2)^ac^ | 0.3 (0.1-0.5)^a^ | 4 | 2.7 (2.3-3.2)^ac^ | 0.5 (0.2-0.7)^ab^ |
|  | **O2** | 4 | 2.9 (2.7-3.2)^ac^ | 0.7 (0.6-0.9)^a^ | 5 | 2.6 (2.3-2.9)^ac^ | 0.4 (0.3-0.5)^a^ | 4 | 2.6 (2.3-2.9)^b^ | 0.4 (0.3-0.5)^a^ |
|  | **O3** | 19 | 2.7 (2.5-2.9)^bcd^ | 0.7 (0.7-0.8)^a^ | 7 | 2.6 (2.3-2.9)^ac^ | 0.6 (0.5-0.6)^b^ | 4 | 2.6 (2.3-2.9)^b^ | 0.6 (0.5-0.6)^b^ |
|  | **O4** | 3 | 2.7 (2.4-3.2)^c^ | 0.7 (0.6-0.8)^a^ | 5 | 2.9 (2.3-3.5)^ac^ | 0.6 (0.6-0.7)^b^ | 3 | 2.9 (2.3-3.5)^c^ | 0.7 (0.4-0.9)^b^ |
| *Sparrows* | **O1** | 9 | 2.9 (2.7-3.1)^d^ | 0.6 (0.5-0.6)^b^ | 7 | 3.3 (2.9-3.7)^d^ | 0.6 (0.5-0.7)^c^ | 15 | 3.3 (2.9-3.7^d^ | 0.5 (0.5-0.6)^a^ |
|  | **O2** | 15 | 3.1 (2.9-3.3)^d^ | 0.7 (0.7-0.8)^ab^ | 8 | 2.9 (2.6-3.3)^e^ | 0.6 (0.6-0.7)^c^ | 12 | 2.9 (2.6-3.3)^d^ | 0.6 (0.5-0.7)^ae^ |
|  | **O3** | 28 | 3.1 (2.9-3.3)^d^ | 0.8 (0.8-0.9)^b^ | 11 | 3.1 (2.7-3.5)^de^ | 0.8 (0.7-0.8)^d^ | 25 | 3.1 (2.7-3.5)^d^ | 0.7 (0.7-0.8)^de^ |
|  | **O4** | 8 | 2.9 (2.6-3.2)^d^ | 0.7 (0.5-0.8)^ab^ | 7 | 3.1 (2.7-3.4)^de^ | 0.7 (0.6-0.9)^d^ | 15 | 3.1 (2.7-3.4)^d^ | 0.7 (0.6-0.8)^d^ |
| *Rats* | **U1** | 3 | 2.8 (2.4-3.3)^abcd^ | 0.6 (0.5-0.7)^a^ | 4 | 2.3 (2.1-2.8)^b^ | 0.3 (0.2-0.4)^b^ | 3 | 2.3 (2.1-2.8)^c^ | 0.5 (0.1-0.9)^ab^ |
|  | **U2** | 17 | 2.8 (2.6-3.0)^c^ | 0.7 (0.6-0.9)^a^ | 8 | 2.6 (2.2-3.1)^c^ | 0.5 (0.4-0.6)^b^ | 3 | 2.6 (2.2-3.1)^c^ | 0.5 (0.1-0.9)^ab^ |
|  | **U3** | 3 | 2.9 (2.5-3.2)^abcd^ | 0.6 (0.6-0.7)^a^ | 3 | 2.5 (2.1-3.4)^abc^ | 0.4 (0.1-0.8)^b^ | 6 | 2.5 (2.1-3.4)^c^ | 0.4 (0.3-0.6)^ac^ |
| *Sparrows* | **U1** | 20 | 2.9 (2.7-3.1)^d^ | 0.7 (0.2-0.9)^a^ | 11 | 2.9 (2.3-3.7)^e^ | 0.6 (0.5-0.7)^cd^ | 17 | 2.9 (2.3-3.7)^d^ | 0.5 (0.4-0.6)^a^ |
|  | **U2** | 7 | 2.8 (2.5-3.2)^d^ | 0.6 (0.5-0.7)^a^ | 21 | 3.0 (2.4-3.5)^e^ | 0.7 (0.6-0.8)^d^ | 35 | 3.0 (2.4-3.5)^d^ | 0.7 (0.6-0.8)^d^ |
|  | **U3** | 21 | 3.0 (2.8-3.2)^d^ | 0.7 (0.5-0.9)^a^ | 15 | 3.0 (2.5-4.3)^de^ | 0.5 (0.4-0.7)^cd^ | 23 | 3.0 (2.5-4.3)^d^ | 0.4 (0.3-0.5)^ad^ |

TP was different between marsh rice rats and seaside sparrows (ranging between 2.3 to 3.3). Where differences occurred between rats and sparrows, rats always occupied lower TP than sparrows. For both species, variation in TP within sites was generally lower in 2015 and 2017 relative to 2016. Seaside sparrow TP did not differ between oiled and unoiled sites in 2015 and few pairwise differences in TP were observed in other years. Specifically, Seaside sparrows had higher TP at O1 (3.3) relative to U1 (2.3) in 2016 (Table S3), and higher TP at O1 (2.7) relative to both U2 (3.0) and U3 (3.0) in 2017. Marsh rice rat TP did not differ between oiled and unoiled sites in 2017, though TP was higher at O1 (3.1) relative to U2 in 2015, and at O4 (3.1) relative to U3 (2.5) in 2016.

The use of terrestrial relative to aquatic resources (α) by seaside sparrows ranged from 30-80% across site and year combinations. Apart from sites U1, U2 and U3 in 2017, seaside sparrows consistently relied on terrestrial resources to a greater degree than aquatic resources (i.e. mode α > 50%). The use of terrestrial relative to aquatic resources (α) by marsh rice rats ranged from 30-70% across site and year combinations. While there was variation at the site level, α was generally lower in marsh rice rats in 2016 relative to 2015 and 2017. While the use of terrestrial relative to aquatic resources (α) did not differ between seaside sparrow and marsh rice rats, when differences occurred (in 2016 and 2017) marsh rats almost always incorporated more aquatic resources relative to seaside sparrows at the same site.
