# Supplementary material for: Stable isotope analyses identify trophic niche partitioning between sympatric terrestrial vertebrates in coastal saltmarshes with differing oiling histories
Source: PeerJ. 2021 Jul 16;9:e11392. doi: 10.7717/peerj.11392 (PMC8288111; doi:10.7717/peerj.11392)
Supplement: Supplemental Information 2 — All values in bold denote significant differences (PP > 0.95). What is the probability that consumer in column 1 has a posterior trophic position less than or equal to consumer in corresponding rows. [file peerj-09-11392-s002.docx]

|  |  | *O. palustris* | *O. palustris* | *A. maritima* | *A. maritima* |
| --- | --- | --- | --- | --- | --- |
|  | Oiling history | Oiled | Unoiled | Oiled | Unoiled |
| **2015** |  |  |  |  |  |
| *O. palustris* | oiled |  | 0.390 | **0.997** | 0.912 |
| *O. palustris* | unoiled | 0.610 |  | **0.999** | 0.948 |
| *A. maritima* | oiled | 0.003 | 0.001 |  | 0.091 |
| *A. maritima* | unoiled | 0.088 | 0.052 | 0.909 |  |
| **2016** |  |  |  |  |  |
| *O. palustris* | oiled |  | 0.073 | **1.000** | **0.998** |
| *O. palustris* | unoiled | 0.927 |  | **1.000** | **1.000** |
| *A. maritima* | oiled | 0.000 | 0.000 |  | 0.069 |
| *A. maritima* | unoiled | 0.002 | 0.000 | 0.931 |  |
| **2017** |  |  |  |  |  |
| *O. palustris* | oiled |  | 0.341 | 1.000 | **0.998** |
| *O. palustris* | unoiled | 0.659 |  | 1.000 | **1.000** |
| *A. maritima* | oiled | 0.000 | 0.000 |  | 0.124 |
| *A. maritima* | unoiled | 0.002 | 0.000 | 0.876 |  |
