# Supplementary material for: Stable isotope analyses identify trophic niche partitioning between sympatric terrestrial vertebrates in coastal saltmarshes with differing oiling histories
Source: PeerJ. 2021 Jul 16;9:e11392. doi: 10.7717/peerj.11392 (PMC8288111; doi:10.7717/peerj.11392)
Supplement: Supplemental Information 3 — All values in bold denote significant differences (PP > 0.95). What is the probability that consumer in column 1 has a posterior trophic position less than or equal to consumer in corresponding rows. [file peerj-09-11392-s003.docx]

|  |  | *O. palustris* | *O. palustris* | *A. maritima* | *A. maritima* |
| --- | --- | --- | --- | --- | --- |
|  | Oiling history | Oiled | Unoiled | Oiled | Unoiled |
| **2015** |  |  |  |  |  |
| *O. palustris* | oiled |  | 0 | 0.656 | 0.008 |
| *O. palustris* | unoiled | **1** |  | **1** | 0.784 |
| *A. maritima* | oiled | 0.344 | 0 |  | 0.006 |
| *A. maritima* | unoiled | **0.992** | 0.216 | 0.994 |  |
| ***2016*** |  |  |  |  |  |
| *O. palustris* | oiled |  | 0.182 | **1** | **1** |
| *O. palustris* | unoiled | 0.818 |  | **1** | **0.999** |
| *A. maritima* | oiled | 0 | 0 |  | 0.2 |
| *A. maritima* | unoiled | 0 | 0.001 | 0.8 |  |
| ***2017*** |  |  |  |  |  |
| *O. palustris* | oiled |  | 0.156 | **0.997** | 0.865 |
| *O. palustris* | unoiled | 0.844 |  | 1 | **0.986** |
| *A. maritima* | oiled | 0.003 | 0 |  | 0.012 |
| *A. maritima* | unoiled | 0.135 | 0.014 | **0.988** |  |
