# Supplementary material for: Stable isotope analyses identify trophic niche partitioning between sympatric terrestrial vertebrates in coastal saltmarshes with differing oiling histories
Source: PeerJ. 2021 Jul 16;9:e11392. doi: 10.7717/peerj.11392 (PMC8288111; doi:10.7717/peerj.11392)
Supplement: Supplemental Information 4 — All values in bold denote significant differences (PP > 0.95). What is the probability that consumer in column 1 has a posterior trophic position less than or equal to consumer in corresponding rows. [file peerj-09-11392-s004.docx]

|  |  | **Species B** |  |  |  |
| --- | --- | --- | --- | --- | --- |
| **Species A** |  | *O. palustris* | *O. palustris* | *A. maritima* | *A. maritima* |
|  | Oiling history | Oiled | Unoiled | Oiled | Unoiled |
| **2015** |  |  |  |  |  |
| *O. palustris* | Oiled |  | 0.003 | **0.983** | **0.955** |
| *O. palustris* | Unoiled | **0.997** |  | **1.000** | **1.000** |
| *A. maritima* | Oiled | 0.017 | 0.000 |  | 0.397 |
| *A. maritima* | Unoiled | 0.046 | 0.000 | 0.603 |  |
| **2016** |  |  |  |  |  |
| *O. palustris* | Oiled |  | 0.030 | 0.006 | 0.463 |
| *O. palustris* | Unoiled | **0.970** |  | 0.508 | **0.978** |
| *A. maritima* | Oiled | **0.994** | 0.492 |  | **0.996** |
| *A. maritima* | Unoiled | 0.537 | 0.023 | 0.004 |  |
| **2017** |  |  |  |  |  |
| *O. palustris* | Oiled |  | 0.007 | 0.412 | **0.955** |
| *O. palustris* | Unoiled | **0.993** |  | **0.994** | **1.000** |
| *A. maritima* | Oiled | 0.588 | 0.007 |  | **1.000** |
| *A. maritima* | Unoiled | 0.045 | 0.000 | 0.001 |  |
